# Long term results of down-staging and liver transplantation for patients with hepatocellular carcinoma beyond the conventional criteria

**DOI:** 10.1038/s41598-019-40543-4

**Published:** 2019-03-07

**Authors:** Matteo Ravaioli, Federica Odaldi, Alessandro Cucchetti, Franco Trevisani, Fabio Piscaglia, Vanessa De Pace, Valentina Rosa Bertuzzo, Flavia Neri, Rita Golfieri, Alberta Cappelli, Antonietta D’Errico, Matteo Cescon, Massimo Del Gaudio, Guido Fallani, Antonio Siniscalchi, Maria Cristina Morelli, Francesca Ciccarese, Maria Di Marco, Fabio Farinati, Edoardo Giovanni Giannini, Antonio Daniele Pinna

**Affiliations:** 1 Department of General Surgery and Transplantation, S. Orsola - Malpighi Hospital, University of Bologna, Bologna, Italy; 2 Department of Medical and Surgical Sciences, Semeiotica Medica Unit, S. Orsola - Malpighi Hospital, University of Bologna, Bologna, Italy; 3 Division of Internal Medicine, Department of Digestive Disease and Internal Medicine, S. Orsola -Malpighi Hospital, University of Bologna, Bologna, Italy; 4 Department of Digestive Disease and Internal Medicine, Radiology Unit, S. Orsola - Malpighi Hospital, Bologna, University of Bologna, Bologna, Italy; 5 Department of Specialised, Experimental and Diagnostic Medicine, Pathology Unit, S. Orsola -Malpighi Hospital, University of Bologna, Bologna, Italy; 6 Department of Anesthesia and Intensive Care, Division of Anesthesiology, S. Orsola - Malpighi Hospital, University of Bologna, Bologna, Italy; 7 Division of Surgery, Policlinico San Marco, Zingonia, Ostio Sotto, Bergamo, Italy; 8 Division of Medicine, Ospedale Bolognini, Seriate, Bergamo, Italy; 9 Department of Surgery and Gastroenterological Sciences, University of Padova, Padova, Italy; 10 Department of Internal Medicine, Gastroenterology Unit, IRCCS - Azienda Ospedaliera Universitaria San Martino, Genova, Italy

## Abstract

The objective of the study is to evaluate 10 years of down-staging strategy for liver transplantation (LT) with a median follow-up of 5 years. Data on long-term results are poor and less information is available for hepatocellular carcinoma (HCC) non-responder patients or those ineligible for down-staging. The outcome of 308 HCC candidates and the long-term results of 231 LTs for HCC performed between 2003 and 2013 were analyzed. HCCs were divided according to tumor stage and response to therapy: 145 patients were T2 (metering Milan Criteria, MC), 43 were T3 successfully down-staged to T2 (Down-Achieved), 20 were T3 not fully down-staged to T2 (Down-not Achieved), and 23 patients were T3 not receiving down-staging treatments (No-Down). The average treatment effect (ATE) of LT for T3 tumors was estimated using the outcome of 535 T3 patients undergoing non-LT therapies, using inverse probability weighting regression adjustment. The 24-month drop-out rate during waiting time was significantly higher in the down-staging groups: 27.6% vs. 9.2%, p < 0.005. After LT, the tumor recurrence rate was significantly different: MC 7.6%, Down-Achieved 20.9%, Down-not Achieved 31.6%, and No-Down 30.4% (p < 0.001). The survival rates at 5 years were: 63% in Down-Achieved, 62% in Down-not Achieved, 63% in No-Down, and 77% in MC (p = n.s.). The only variable related to a better outcome was the effective down-staging to T2 at the histological evaluation of the explanted liver: recurrence rate = 7.8% vs. 26% (p < 0.001) and 5-year patient survival = 76% vs. 67% (p < 0.05). The ATE estimation showed that the mean survival of T3-LT candidates was significantly better than that of T3 patients ineligible for LT [83.3 vs 39.2 months (+44.6 months); p < 0.001]. Long term outcome of T3 down-staged candidates was poorer than that of MC candidates, particularly for cases not achieving down-staging. However, their survival outcome was significantly better than that achieved with non-transplant therapies.

## Introduction

The Milan Criteria (MC), proposed in 1996^1^, have paved the way for liver transplantation (LT) in patients with hepatocellular carcinoma (HCC). The selection of patients with a solitary tumor ≤5 cm or up to 3 tumors with the largest being ≤3 cm in diameter, allows a 5-year survival of 70% and a recurrence rate of only 10%, thus becoming comparable to outcomes achievable in non-oncological indications for LT^1,2^, and accomplishing the concept of “equity”.

However, MC precludes access to LT of some patients with a potentially good outcome, and many groups have investigated how these criteria can be expanded, without affecting patient survival and tumor recurrence^3,4^. One possibility is to expand the criteria based on the slightly extended tumor burden, an example of this strategy being the “up-to-seven” rule (the sum of the number of nodules plus the size in cm of the largest one cannot exceed seven), derived from a large retrospective database^5^. Another way to expand the criteria for transplantation relies on the selection of less aggressive tumors responding to the treatment, the so called down-staging strategy. This opens the LT doors to patients with HCC outside MC (but within predefined maximum tumor burdens) who have been variously but successfully treated in order to reduce the tumor burden and, possibly, re-entering the framework of MC. In 2002, our group designed a down-staging protocol based on different neoadjuvant treatments aimed at bringing the tumor back within MC limits, which was prospectively applied in our clinical practice. According to an intention-to-treat analysis, the LT outcome of down-staged patients was found to be comparable to that of recipients originally meeting the MC in terms of 3-year survival^6^.

Based on the good results obtained in this exploratory study and by another study by the University of California San Francisco (UCSF) where a quite similar strategy was adopted^7^, a recent international consensus conference on LT for HCC addressed this issue publishing the following recommendations: “LT can be considered after successful down-staging, the survival of successfully down-staged patients should be comparable to HCC patients within MC from the beginning. The criteria for successful down-staging should include tumor size and number of active lesions, although no common agreement is reached on the threshold”^8^.

The UCSF group updated their population study after a median follow-up of more than 3 years and confirmed its previous results, although some categories of patients (4–5 lesions of HCCs) did not have definitive evidence due to the small number of cases^9^. Moreover, open questions remained about the patients transplanted without successful down-staging or not suitable for down-staging^10^.

Since post-transplant HCC recurrence may occur even many years after surgery and recurrent cases may survive for more than 1–2 years^11,12^, how much such recurrences can impair the long-term success of down-staging strategies has not been defined. In fact, prospective long-term results of LT performed after down-staging procedures are limited.

In order to give further insight on the long-term results of LT performed in the setting of HCC down-staging, the present study analyses our ten years’ experience, including a follow-up of 5 years post-LT.

## Materials and Methods

### Study population and bridge treatment procedures

The study includes 308 cases with HCC listed for LT in our center between 2003 and 2013 and the median post-LT follow-up was 5 years. The series we described in 2008^6^ was therefore updated and all new consecutive cases were added. The local medical ethics committee of Sant’Orsola-Malpighi Hospital - University of Bologna approved this retrospective observational study and informed consent was signed by enrolled patients.

The diagnosis of HCC was performed according to international guidelines recommendations released in the corresponding period of diagnosis^13,14^. When the imaging work up led to dubious results, a biopsy was performed on the target lesion. The retrospective review of all the lesions confirmed that they were LIRAD 5 nodules^15^.

### Down-staging protocol

- The criteria approved by the Bologna liver transplant Committee were: single HCC ≤8 cm; bifocal HCC, each ≤5 cm; multiple HCC with ≤5 nodules, each ≤4 cm and a total tumor diameter ≤12 cm.
- All the cases had to meet MC at the end of the down-staging procedures, including both newly developed nodules and those still active, the diameter of which was calculated including the necrotic part. The total number of nodules, treated or not treated, and present at the beginning or developing later, could not exceed the limit approved for the protocol.
- The type of neoadjuvant treatment was decided case-by-case and by a multidisciplinary team including hepatologists, liver surgeons, radiologists and pathologists, following the available guidelines and our previous experience^6,13,14,16–18^.
- The possible options were: liver resection (LR), transarterial chemo-embolization (TACE), percutaneous ethanol injection (PEI), percutaneous radiofrequency ablation (RFA). The cases of liver resection with radical intent, with no evidence of disease and R0 were not included in the waiting list and in the down-stage group.
- After successful down-staging, a minimum follow-up of 3 months of permanence within the MC without additional treatments was considered necessary to include patients in the waiting list.
- Alpha-fetoprotein level (AFP) had to remain <400 ng/dL during the waiting time.
- Macro-vascular or biliary invasion by the tumor were exclusion criteria.

All listed patients were followed-up with ultrasonography (US) or computed tomography (CT) every 3 months. At the follow-up, the size of active nodules (presenting contrast enhancement at imaging) was measured, also including the necrotic portion. Both down-staged active nodules and new nodules that appeared during the waiting time were considered in establishing whether the MC were met. At any time of the waiting list period, the total number of nodules (treated or not, present at the beginning or developing subsequently) could not exceed the number approved in the protocol.

The listed patients with HCC received priority by adding extra points to the biochemical MELD (model for end stage liver disease) score, according to their tumor stage and the waiting time, as previously reported^16,17^.

### Exceptions to the down-staging protocol

After 2006, our Scientific Committee decided to also evaluate cases meeting our down-staging criteria, but unable to be effectively down-staged at imaging evaluation or not amenable to down-staging due to impaired liver function. These patients were discussed case-by-case during the multidisciplinary meeting and selected for LT in order to explore the outcome of these new HCC categories with respect to our previous study.

The study was approved by the local institutional review committee. All the procedures were performed upon approval by our ethics committee and collection of informed consent from all the patients.

### Study groups

Following the down-staging protocol and the exceptions, four groups were created on the basis of the pre-operative imaging:

1. 186 HCCs always meeting the conventional (Milan) criteria (T2), who received, or not, pre-operative bridge treatments (MC);
2. 65 HCCs initially exceeding the conventional criteria (T3), but meeting the criteria for enrolment in our down-staging protocol and who were successfully down-staged to T2 at the last pre-LT imaging (Down-Achieved);
3. 30 HCCs initially not meeting the conventional criteria (T3), but within the criteria for enrolment in our down-staging protocol and who failed to achieve a down-staging to T2 at the last pre-LT imaging (Down-not Achieved);
4. 27 HCCs exceeding the conventional criteria (T3), but within the criteria for the enrolment in our down-staging protocol and who did not receive any antitumor down-staging therapy, due to their impaired liver function (No-Down).

### Statistical analysis

Fisher’s exact test, Mann-Whitney test or Chi-square test were applied, as appropriate. The intention to treat the survival of patients was calculated by the Kaplan-Meyer method, starting from the date of enrolment to the date of death or the most recent follow-up visit. The post-transplant survival was calculated by the Kaplan-Meyer method, starting from the date of LT to the date of death or the most recent follow-up visit^6^. The difference between survivals was tested by the Log-rank test. The follow-up started in January 2003 and ended in December 2016. Statistical analysis was performed with SPSS (SPSS Base 10.0; Application Guide, SPSS Inc., Chicago, IL, 1998).

### Statistical analysis to compare the efficacy of liver transplantation vs. other treatments

The Italian Liver Cancer (ITA.LI.CA) database of 2014 was used to compare the outcomes of the present study population with a population in which transplantation was not performed. From this prospective registry of HCC patients consecutively diagnosed and followed by 24 Italian medical institutions, only patients with complete data and with a T3 tumor were retained for comparison with the current T3 candidates. In December 2014, the ITA.LI.CA data set included 6581 HCC patients. From these, according to the selection criteria of our down-staging protocol, 742 HCC-T3 patients not treated with LT were retained.

Cumulative incidences of the competing events of interest were calculated using the Fine and Gray competing risks approach using the STATA syntax stcrreg (StataCorp. Stata Statistical Software: Release 12.). When necessary, factors identified having a p < 0.10 on simple (univariate) competing risk analysis were entered into a multivariable regression model. A p-value < 0.05 was considered statistically significant in all the analyses.

### Statement of Ethics

Demographic, clinical, laboratory and imaging data of study patients were collected and analyzed in accordance with the Helsinki Declaration and Regional Ethics Committee under informed consent of the patients.

## Results

### Pre- and post-LT features of the study population

Pre-operative clinical features were comparable among the four groups except for liver function, which was worst in the No-Down group (Table 1). As expected, the number and mean size of the nodules were different among groups. TACE was the most frequently adopted neo-adjuvant treatment, especially for patients undergoing the down-staging protocol. The histological evaluation of explanted livers showed no cases with macro-vascular tumor invasion, and the rate of complete tumor necrosis did not differ among the groups (Table 2). As expected, the Down-not Achieved and the No-Down groups more frequently showed a T3 stage or higher (≥4 nodules of any size) and histological features of tumor aggressiveness, such as micro-vascular invasion and moderate/poor tumor differentiation.

**Table 1:** Clinical features of patients on the waiting list divided according to the study population.

	Meeting Bologna down-staging criteria (n = 122)			T2 group MC	P
	Down-staging completed	Down- staging not completed	Down- staging not performed		
	Down-Achieved	Down-No Achieved	No-Down		
	(n = 65)	(n = 30)	(n = 27)	(n = 186)	
Male, n (%)	60 (92.3)	26 (86.7)	***18 (66***.***7)***	160 (86.0)	***p*** = ***0***.***02***
Age y, mean ± SD (median)	56 ± 9 (57)	55 ± 11 (57)	56 ± 7 (57)	57 ± 8 (59)	p = 0.3
Liver disease HCV + , n (%)	36 (55.5)	21 (70.0)	18 (66.7)	109 (58.6)	p = 0.1
CHILD C, n (%)	13 (20.0)	5 (16.7)	***15 (55***.***6)***	44 (23.8)	***p*** = ***0***.***001***
MELD, mean ± SD (median)	13 ± 6 (11)	13 ± 5 (14)	16 ± 6 (17)	13 ± 5 (13)	***p*** = ***0***.***05***
AFP ng/dl median	9	10	10	10	p = 0.37
Waiting time median days	362	180	141	285	p = 0.7
Single HCC, n (%)	6 (9.2)	0	***14 (51***.***9)***	***96 (51***.***6)***	***p*** = ***0***.***001***
2 HCC, n (%)	16 (24.6)	9 (30.0)	8 (29.6)	51 (27.4)	
3 HCC, n (%)	11 (16.9)	3 (10.0)	5 (18.5)	39 (19.9)	
4 HCC, n (%)	15 (23.1)	14 (46.7)	—	—	
5 HCC, n (%)	17 (26.1)	4 (13.3)	—	—	
1 HCC mean size ± SD (median)	3.7 ± 1.6 (4)	3.6 ± 1.3 (3.9)	2.8 ± 1.1	2.5 ± 0.9	***p*** = ***0***.***001***
2 HCCs mean size ± SD (median)	2 ± 1 (1.9)	2.3 ± 0.9 (2.3)	1.3 ± 1.2	1.1 ± 0.9	***p*** = ***0***.***001***
3 HCCs mean size ± SD (median)	1.4 ± 1.1 (1.2)	1.4 ± 1.1 (1.1)	0.8 ± 0.7	0.8 ± 0.7	***p*** = ***0***.***001***
4 HCCs mean size ± SD (median)	1.1 ± 0.8 (1)	0.9 ± 0.7 (1)	—	—	***p*** = ***0***.***001***
5 HCCs mean size ± SD (median)	1 ± 1.8 (1)	0.8 ± 0.5 (0.6)	—	—	***p*** = ***0***.***001***
Pre-LT Treatment, n (%)	65 (100)	30 (100)	21 (77.8)	167 (89.8)	***p*** = ***0***.***001***
TACE, n (%) mean ± SD procedure	52 (80.0) 1.7 ± 1.2	28 (93.3) 1.7 ± 1.3	18 (66.7) 1.7 ± 1.0	118 (63.4) 1.8 ± 1.0	***p*** = ***0***.***002***
PEI mean ± SD procedure	19 (29.2) 1.2 ± 0.4	8 (26.7) 1.1 ± 0.4	3 (11.1) 1.0 ± 0	37 (19.9) 1.2 ± 1.0	p = 0.19
RF mean ± SD procedure	28 (43.1) 1.5 ± 1.3	9 (30.0) 0.9 ± 0.3	8 (29.6) 1.1 ± 0.4	65 (34.9) 1.3 ± 0.9	p = 0.49
LR for down-stage	18 (27.7)	4 (13.8)	—	—	**p** = **0**.**001**
Previous R0 LR	—	—	3 (11.1)	26 (14.1)	**P** = **0**.**005**

**Table 2:** Explant histological results divided according to the study population.

	Meeting Bologna down-staging criteria (n = 86)			T2 group MC	P
	Down- staging completed	Down- staging not completed	Down- staging not performed		
	Down-Achieved	Down-not Achieved	No-Down		
	(n = 43)	(n = 20)	(n = 23)	(n = 145)	
≥ T3, n (%)	16 (37.2)	15 (75.0)	17 (73.9)	32 (22.1)	***p*** = ***0***.***001***
Grade of differentiation Well, n (%)	18 (42)	3 (15)	6 (26)	83 (57)	***p*** = ***0***.***001***
Moderate-Poor, n (%)	25 (58)	17 (75)	17 (74)	62 (43)	
Microvascular tumor invasion, n (%)	22 (51.2)	14 (70.0)	15 (65.2)	41 (28.3)	***p*** = ***0***.***001***
Complete necrosis, no viable tumor, n (%)	5 (11.6)	2 (10.0)	2 (8.7)	20 (13.8)	p = 0.88

### Risk of drop-out during waiting time

**Figure 1:**
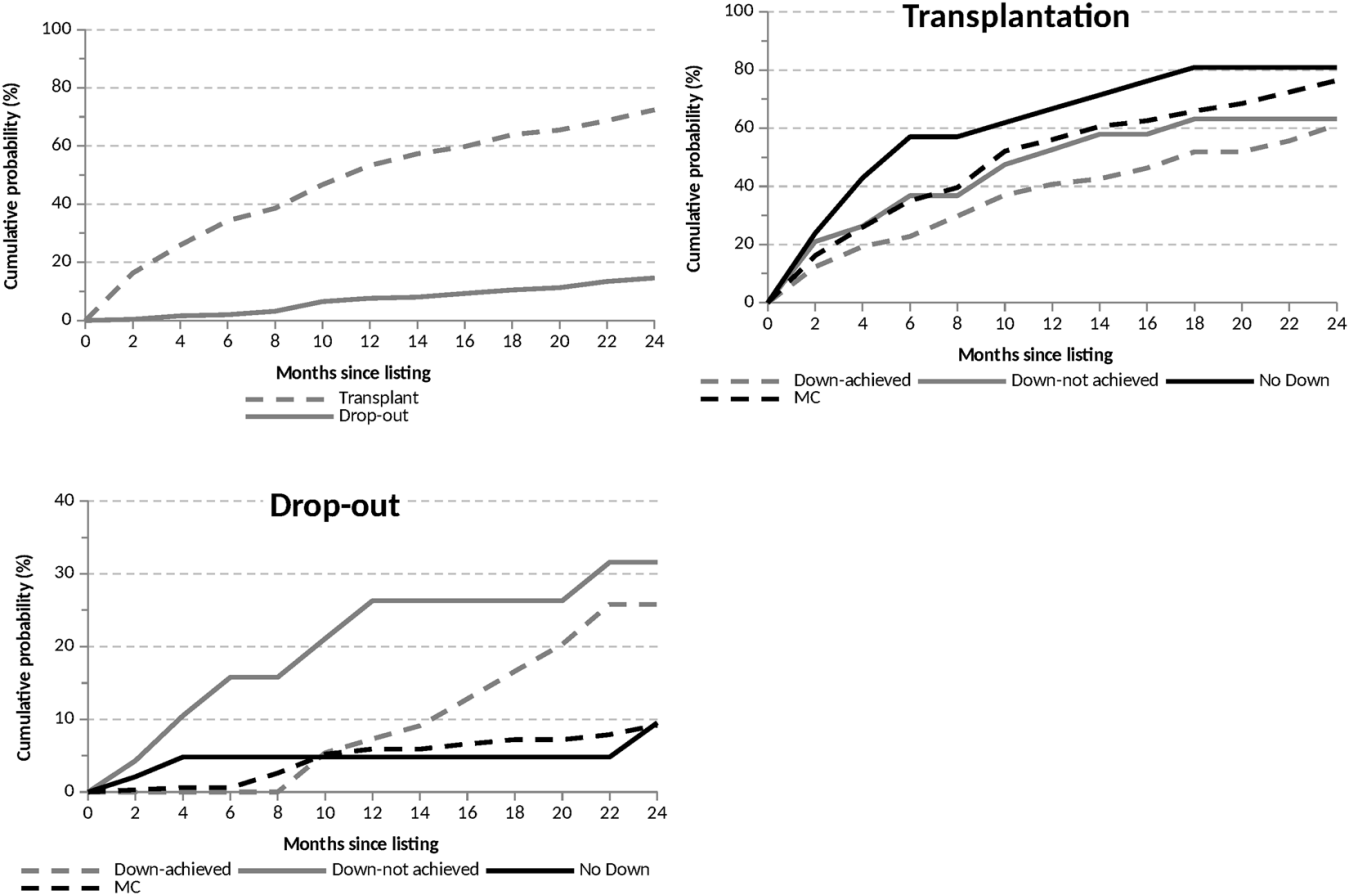
Competing risk analysis of the probability of drop-out and transplantation in the study groups.

In the whole population, the LT rate at 24 months was 75% and the drop-out rate during the waiting time 18%. The competing-risk analysis showed a higher risk of drop-out for Down-Achieved and Down-not Achieved compared to MC and No-Down, whereas predicted probabilities of LT remained roughly similar among the four groups (Fig. 1). In particular, the pooled predicted drop-out rate at 24 months was 27.6% for Down-Achieved and Down-not Achieved patients, a figure significantly higher than that of MC and No-Down patients (9.2%, p < 0.001). Multivariate competing-risks analysis showed that the study group was the only significant variable associated with the exclusion from LT.

The median waiting time was not significantly different among the groups, as reported in Table 1, but considering the waiting time longer than 6 months, it was differently distributed (79% Down-Achieved, 47% Down-not Achieved, 38% days No-Down and 64% MC, p < 0.005). The waiting time longer than 6 months was also statistically related to a higher rate of drop-out on the waiting list (8% vs. 26%, p < 0.001).

### Recurrence rate, post-LT survival and intention to-treat survival

**Figure 2:**
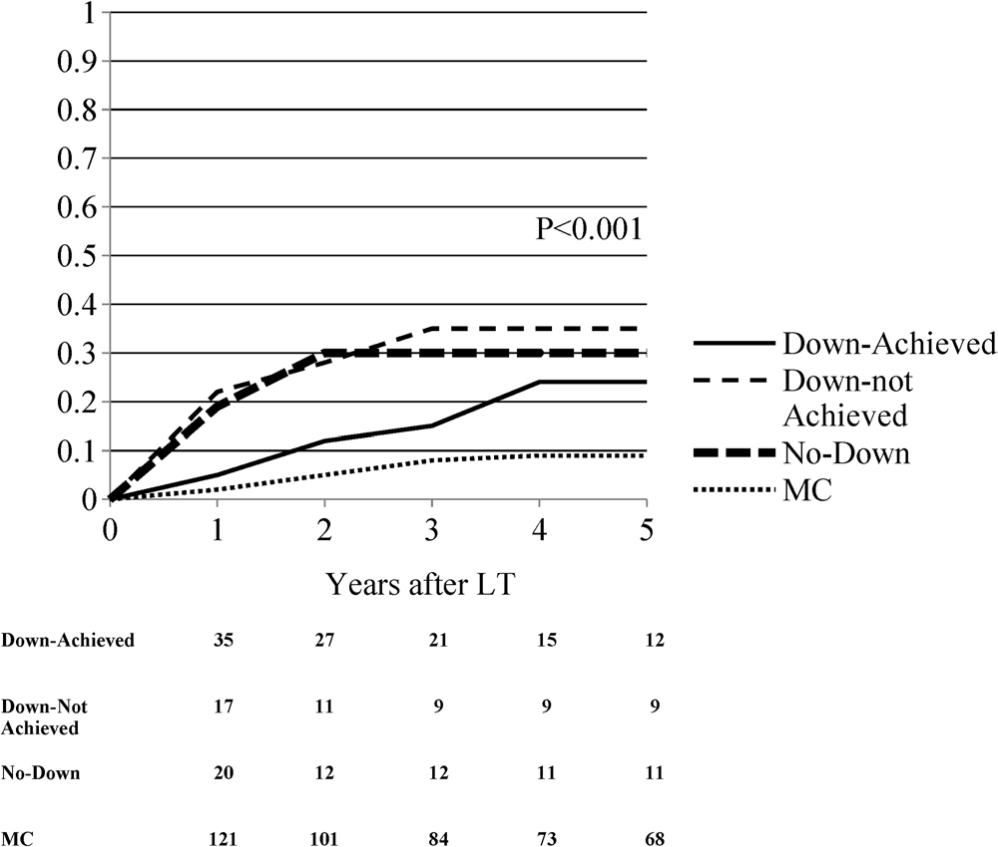
Kaplan-Meier probabilities of tumor recurrence by study populations.

After a median follow-up of 60 months, the post-LT overall recurrence rate was significantly different among groups, being 7.6% in MC, 20.9% in Down-Achieved, 31.6% in Down-not Achieved and 30.4% in No-Down patients (p < 0.001) (Fig. 2).

**Figure 3:**
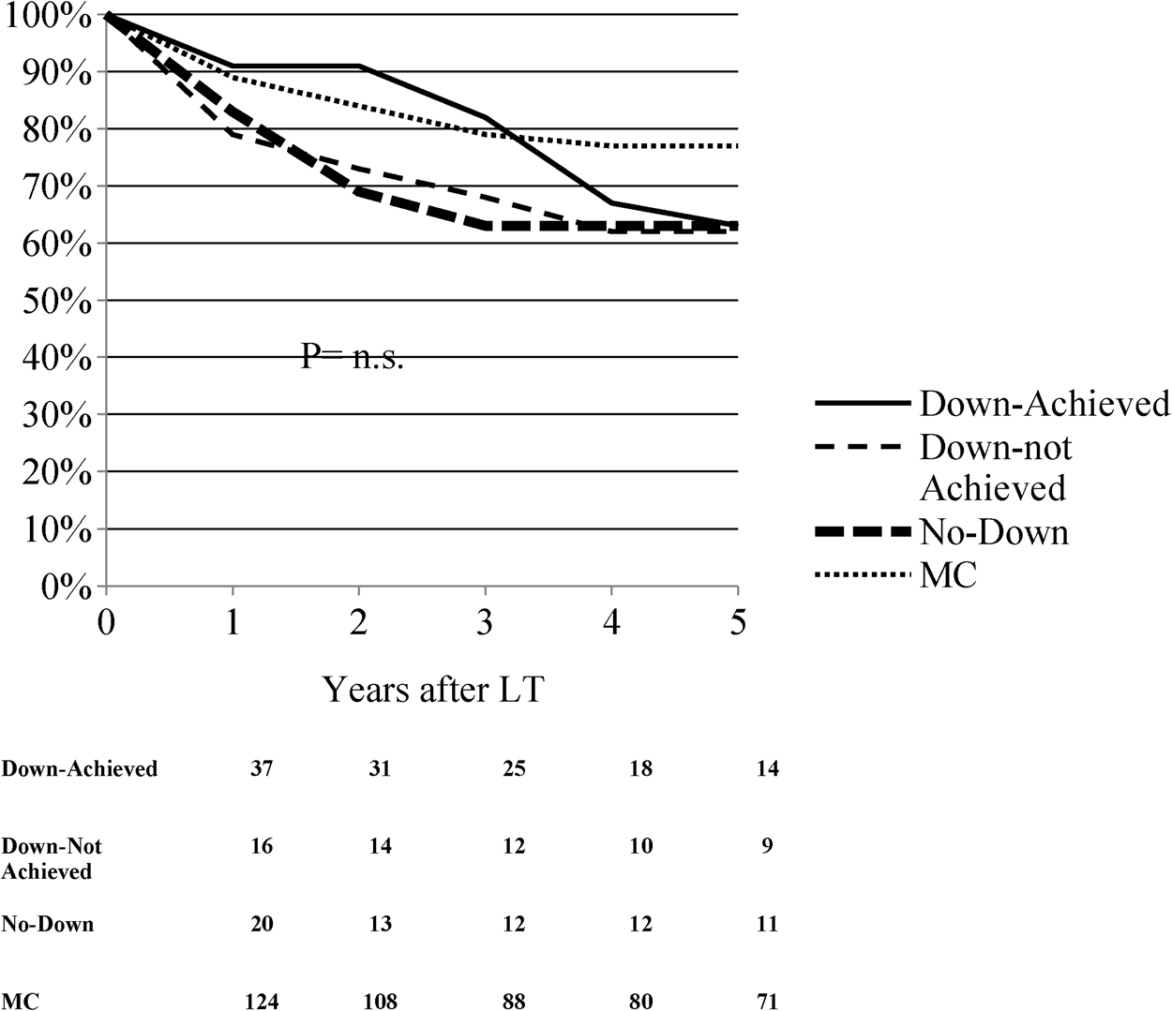
Kaplan- Meier probabilities of patient survival by study populations.

The 3-year overall survival of MC and Down-Achieved patients was 80%, better than in the other two groups. However, in Down-Achieved patients it dropped to about 60% at 5 years, becoming similar to the survival rates of Down-not Achieved and No-Down patients (Fig. 3). In particular, survival rates at 5 years were: 63% in Down-Achieved, 62% in Down-not Achieved, 63% in No-Down, and 77% in MC patients (p = n.s.). The only variable related to a lower recurrence rate and a better survival was the effective down-staging to T2 at the histological evaluation of the explanted liver: recurrence rate = 7.8% vs. 26% (p < 0.001) and 5-year patient survival = 76% vs. 67% (p < 0.05). The waiting time longer than 6 months was not related to tumor recurrence and patient survival after LT.

**Figure 4:**
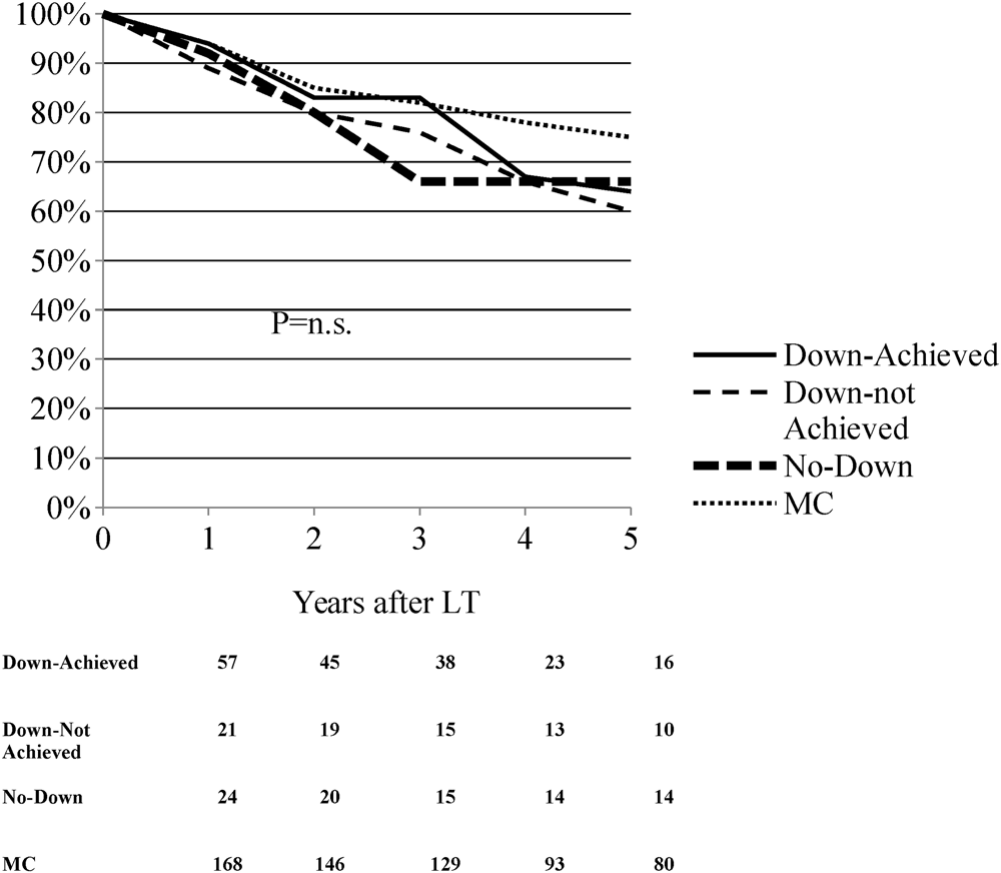
Kaplan-Meier probabilities of intention-to-treat survival by study populations.

The intention-to-treat analysis of survival showed similar results to patient survival after LT, at 3 years the survival of MC and Down-Achieved was equal to 82–83%, but in the last group it dropped to 64% at 5 years, as in the Down-not Achieved and No-Down groups (Fig. 4). The 5-year survival rate among groups did not show statistical differences and was: 64% in Down-Achieved, 60% in Down-not Achieved, 66% in No-Down, and 75% in MC patients (Fig. 4).

### Comparison with ITA.LI.CA T3 patients

**Figure 5:**
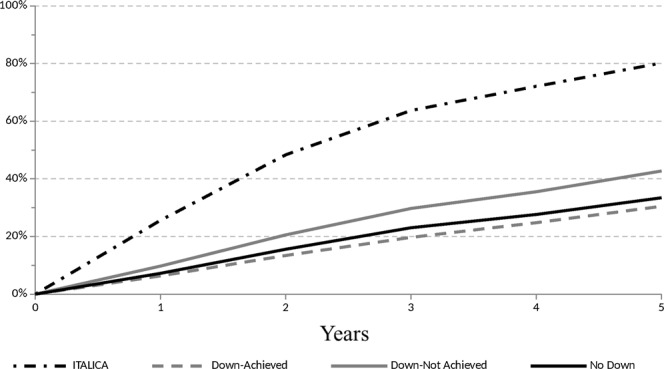
Multivariate competing-risk regression adjusted for covariates Age and MELD score at start and predicted probabilities of death among ITALICA, Down-Achieved, Down-not Achieved and No-Down groups.

Survivals since listing for LT of Down-Achieved, No-Down and Down-not Achieved patients were compared to the outcome of T3 patients from the ITA.LI.CA database. These patients were selected following the criteria of our down-staging protocol. Multivariate competing-risk regression was adjusted for the covariates age and MELD at listing or, for ITA.LI.CA patients, at HCC diagnosis (see Supplementary Material), and predicted probabilities of death were plotted (Fig. 5). Notably, the predicted probability of death at 5 years for ITA.LI.CA T3 patients was 80%, whereas the corresponding figures of Down-Achieved, No-Down and Down-not Achieved patients were 30%, 33% and 43%, respectively (p < 0.001).

## Discussion

The current updated series of patients with a 5-year follow-up after LT showed that the down-staging of HCCs outside the MC criteria had a higher risk of tumor recurrence, but their outcome fulfilled both the “utility”^19^ and the “transplant benefit” principle^18,20^. In fact, their 5-year survival rate was >50% and remarkably higher than that of T3 HCCs treated with therapies other than LT.

Our previously published data, based on a median follow-up of 2.5 years, showed both patient survival and HCC recurrence rate after LT comparable between down-staged and MC patients^6,8^. Similar results were obtained by Yao *et al*. even with a longer follow-up^9^. The study population of Yao *et al*. was quite different from ours in both previous^21^ and present up-dated series. In particular, American patients undergoing down-staging more frequently had less aggressive tumor biology, as testified by the low rate of micro-vascular invasion and a better differentiation degree. Furthermore, they reported a few cases with a number of nodules >4 and a higher rate of complete tumor necrosis.

These differences likely explain why our results were poorer than those reported by Yao *et al*.^9^. A recent series reported by Llovet *et al*.^22^, in which the histological features were quite comparable to our data, showed a similar high rate of tumor recurrence.

Nonetheless, in the Llovet study and in our series a satisfactory 5-years survival after LT was observed.

Many authors, including ourselves, believe that the down-staging procedure makes it possible to select tumors with more favorable biology^6,23^. On the other hand, these data were not so evident in our study, where the histological examination of explanted livers showed a higher rate of micro-vascular tumor invasion and a poor differentiation degree in down-staged patients. Furthermore, the no-Down group, which did not undergo neoadjuvant treatments due to liver failure, had a lower drop-out rate during the waiting time and a satisfactory survival after LT. The shorter waiting time of this group may be compared to the shorter waiting time reported by the recent series with living donor liver transplantation for HCC beyond Milan Criteria^24^.

Therefore, the present study fuels the debated issue of the minimal time to wait until eligibility for LT is achieved, in particular for HCC patients with a high MELD score.

The present report addresses some aspects explored by previous studies^9,25^, i.e. the outcome of HCC patients not treated by down-staging and those treated but not effectively down-staged. The definition of effective down-staging still remains controversial; we used a radiological response able to meet the conventional Milan criteria, but any other radiological response could also be evaluated. These two groups (not treated for down-staging due to high MELD score or treated with down-staging without the expected response) showed a rate of post-LT tumor recurrence close to 30%, which was significantly higher than the recurrence found in cases fulfilling the conventional criteria or effectively down-staged. Nevertheless, the 5-year survival rate was >50% even in these patients, confirming the utility of LT, and the comparison with a cohort of patients with the same tumor stage, but treated without LT, showed a significant benefit^18,20^.

This last result indicates that, although we must pay the “price” of lower survival, LT remains a pursuable therapeutic option for non- or ineffectively-down-staged patients, even in the light of the “equity” standpoint of organ allocation^19^. Moreover, the adoption of expanded criteria for HCC transplant will be favored by the recent advent of highly effective anti- HCV (hepatitis C virus) infection therapies that will decrease of the number of non HCC competing patients, candidates for LT due to liver failure. A word of caution is however needed, as our cases not suitable for down-staging (due to poor liver function), or in whom the procedure was unsatisfactory, fulfilled the Bologna down-staging selection criteria at baseline. Therefore, their acceptable post-LT survival cannot represent what is achievable in all T3 patients, suggesting instead that careful selection of these cases is needed in order to prevent the failure of the “utility” principle accomplishment.

Another important finding picked up thanks to the long follow-up of the current study was the significant increased tumor recurrence rate in patients beyond MC at the histological analysis, regardless of their pre-operative group distribution. This highlights even more the importance of very careful imaging evaluation before LT in order to really identify cases with different risks of poor outcome. So, we stress, as in our previous report^8^, the relevance of up-to-date contrast-enhanced ultrasonography and magnetic resonance (MR) in order to evaluate the response to loco-regional treatments for patients outside the MC, but also for conventional cases who are treated during the waiting time. The discrepancy among radiological and histological findings may increase with the number of nodules: in our series, patients with >3 nodules (33 cases) effectively treated with down-staging and then transplanted, had a low recurrence rate, comparable to the conventional criteria. This group was twice as large as that reported by Yao’s study^9^ and it therefore adds more evidence to the efficacy of down-staging in patients with more than 3 nodules.

Although the prospective design of the down-staging protocol and the long follow-up are the strengths of our study, it suffers from some limitations.

The outcomes were retrospectively evaluated, even if the data were gathered in a prospective manner and they come from a single institution. There were relatively few study cases, mainly since the entire population was dichotomized into 4 groups. Therefore, the reproducibility of our results needs further confirmation. Furthermore, other data are probably necessary for a final conclusion concerning the patients who did not achieve radiological down-staging and were transplanted or those who were transplanted without doing any down-staging procedures due to liver failure. These cases were discussed in a multidisciplinary meeting and the only strict criteria for exclusion were the AFP higher than 400 ng/dL and the absence of macro-vascular or biliary invasion. The AFP limit was probably the key selection for our satisfactory patient survival, as stressed by recent studies^26,27^ and it was the most effective biological marker behavior, available pre-operatively. The many other clinical and pathological variables relevant during the multidisciplinary discussion, like recipient age, history of disease, type of response to treatment, type of donor offered and so on, are still too difficult to include in an algorithm process.

In conclusion, our study measured the price to be paid by transplant patients outside conventional HCC criteria (and within the Bologna criteria) after effective or ineffective down-staging procedures. The long-term outcome of down-staging candidates was poorer than that achievable with the conventional criteria, particularly for cases not meeting the protocol. Nevertheless, it can be considered acceptable since it is much better than that obtained with non-LT treatments.

### Appendix

#### Other members of the ITA.LI.CA Group

Department of Internal Medicine, Gastroenterology Unit, Policlinico San Martino, University of Genova: Alessandro Moscatelli, Gaia Pellegatta, Vincenzo Savarino; Department of Medicine and Surgery Sciences, University of Bologna: Maurizio Biselli, Paolo Caraceni, Marco Domenicali, Annagiulia Gramenzi, Donatella Magalotti, Carla Serra, Laura Venerandi, Marco Zoli; Gastroenterology Unit, University of Bologna, Bologna: Antonio Colecchia, Giovanni Marasco, Federico Ravaioli; Radiology Unit, Department of Diagnostic and Preventive Medicine, University of Bologna, Bologna: Alberta Cappelli, Rita Golfieri, Cristina Mosconi, Matteo Renzulli; Gastrenterology Unit, Belcolle Hospital, Viterbo: Eugenio Caturelli, Paola Roselli, Valentina Lauria, Giorgio Pelecca; Medicine Unit, Viterbo: Serena Dell’Isola, Anna Maria Ialungo, Elena Rastrelli; Department of Internal and Specialistic Medicine, Gatsroenterology Unit, University of di Palermo, Palermo: Calogero Cammà, Simona Attardo, Margherita Rossi, Giulia Cavani; Departemnet of Internal and Specialistic Medicine, Internal Medicine Unit, Ospedali Riuniti Villa Sofia-Cervello Hospital, Palermo: Roberto Virdone, Andrea Affronti; Gastroenterology Unit, Regional Hospital of Bolzano, Bolzano: Andrea Mega; Surgery Unit, Policlinico S. Marco, Zingonia: Paolo Del Poggio, Stefano Olmi; Internal Medicine, Infermi Hospital of Faenza, Faenza: Francesco Giuseppe Foschi, Vittoria Bevilacqua, Anna Chiara Dall’Aglio, Giorgio Ercolani, Erica Fiorini, Andrea Casadei Gardini, Arianna Lanzi, Federica Mirici Cappa; Gastroenterology Unit, Azienda Ospedaliero-Universitaria Pisana, Pisa: Rodolfo Sacco, Valeria Mismas; Clinic of Gastroenterology, University of Marche, Ancona: Gianluca Svegliati Baroni, Laura Schiadà; Department of Gastrointestinal and Surgery Sciences, University of Padova, Padova: Alessia Gazzola, Francesca Murer, Caterina Pozzan, Veronica Vanin; Department of Molecular Medicine, University of Padova, Padova: Luisa Benvegnù; Gatroenterology and Internal Medicine Unit, Complesso Integrato Columbus, Cattolica University of Roma, Roma: Gian Ludovico Rapaccini, Nicoletta de Matthaeis; Gastroenterology and Internal Medicine Unit, Policlinico Gemelli, Cattolica University of Roma, Roma: Antonio Gasbarrini, Emanuele Rinninella; Hepatology and Infective Disease Unit, Azienda Ospedaliero-Universitaria, Parma: Gabriele Missale, Elisabetta Biasini; Medicine Unit, Azienda Ospedaliera Bolognini, Seriate: Claudia Balsamo, Elena Vavassori; Department of Cliinical Medicine and Surgery, Gatroenterology Unit, Federico II University, Napoli: Filomena Morisco, Maria Guarino, Anna Vitiello; Gerardo Nardone; Gatroenterology Unit, Ospedale Sacro Cuore Don Calabria, Negrar: Alberto Masotto, Fabiana Marchetti, Matteo Valerio; Internal Medicine and Hepatology, Department of Sperimental and Clinical Medicine, Firenze: Fabio Marra, Sami Aburas, Claudia Campani, Gabriele Dragoni; Department of Medicine, Internal Medicine and Hepatology, Ospedale Fatebenefratelli, Milano: Franco Borzio.

## Supplementary information

Demographical, cancer, anti-tumoral treatment and survival data of HCC liver recipients.
