# Supplementary material for: Long term results of down-staging and liver transplantation for patients with hepatocellular carcinoma beyond the conventional criteria
Source: Sci Rep. 2019 Mar 7;9:3781. doi: 10.1038/s41598-019-40543-4 (PMC6405768; doi:10.1038/s41598-019-40543-4)
Supplement: Supplementary file 1 — Demographical, cancer, anti-tumoral treatment and survival data of HCC liver recipients. [file 41598_2019_40543_MOESM1_ESM.docx]

**Long term results of down-staging and liver transplantation for patients with hepatocellular carcinoma beyond the conventional criteria**

Matteo Ravaioli^1^, Federica Odaldi^1^, Alessandro Cucchetti^1^, Franco Trevisani^2^, Fabio Piscaglia^3^, Vanessa De Pace^1^, Valentina Rosa Bertuzzo^1^, Flavia Neri^1^, Rita Golfieri^4^, Alberta Capelli^4^, Antonietta D’Errico^5^, Matteo Cescon^1^, Massimo Del Gaudio^1^, Guido Fallani^1^, Antonio Siniscalchi^6^, Maria Cristina Morelli^1^, Francesca Ciccarese^7^, Maria Di Marco^8^, Fabio Farinati^9^, Edoardo Giovanni Giannini^10^, Antonio Daniele Pinna^1^.

^1^ Department of General Surgery and Transplantation, S. Orsola - Malpighi Hospital, University of Bologna, Bologna, Italy.

^2^ Department of Medical and Surgical Sciences, Semeiotica Medica Unit, S. Orsola - Malpighi Hospital, University of Bologna, Bologna, Italy.

^3^ Division of Internal Medicine, Department of Digestive Disease and Internal Medicine, S. Orsola -Malpighi Hospital, University of Bologna, Bologna, Italy.

^4^ Department of Digestive Disease and Internal Medicine, Radiology Unit, S. Orsola - Malpighi Hospital, Bologna, University of Bologna, Bologna, Italy.

^5^ Department of Specialised, Experimental and Diagnostic Medicine, Pathology Unit, S. Orsola -Malpighi Hospital, University of Bologna, Bologna, Italy.

^6^ Department of Anesthesia and Intensive Care, Division of Anesthesiology, S. Orsola - Malpighi Hospital, University of Bologna, Bologna, Italy.

^7^ Division of Surgery, Policlinico San Marco, Zingonia, Ostio Sotto, Bergamo, Italy

^8^ Division of Medicine, Ospedale Bolognini, Seriate, Bergamo, Italy.

^9^ Department of Surgery and Gastroenterological Sciences, University of Padova, Padova, Italy.

^10^ Department of Internal Medicine, Gastroenterology Unit, IRCCS - Azienda Ospedaliera Universitaria San Martino, Genova, Italy.

**Supplemental Information**

*Table:* Demographical, cancer, anti-tumoral treatment and survival data of HCC liver recipients.

| Variable | N = 535 |
| --- | --- |
| Age (year) | 66 (60 – 70) |
| Male | 420 (78.5%) |
| MELD at diagnosis | 10 (8 – 13) |
| AFP (ng/mL) | 23.2 (6.6 – 202) |
| Number of lesions | 2 (1 – 4) |
| Single tumor | 259 (48.4%) |
| 2-3 tumors | 58 (10.8%) |
| More than 3 tumors | 218 (40.7%) |
| Maximum tumor diameter | 4.2 (3.0 – 6.5) |
| Treatment adopted |  |
| TACE/TAE | 273 (51.1%) |
| BSC / Other | 93 (17.3%) |
| Ablation | 69 (12.9%) |
| HR | 80 (14.9%) |
| Sorafenib | 20 (3.8%) |
| Overall survival |  |
| Median (months; 95% C.I.) | 23.1 (20.6 – 25.7) |
| 1-year | 73.6% |
| 3-year | 34.2% |
| 5-year | 18.1% |

ITA.LI.CA. population. Values are reported as median and interquartile range.
